# What Drives the Alien Parrot Richness and Occurrence in Urban Green Spaces along the Annual Cycle in Buenos Aires City, Argentina?

**DOI:** 10.3390/ani13213426

**Published:** 2023-11-06

**Authors:** Sebastián Martín Santiago, Nínive Paes Cavalcante, Lucas Matías Leveau

**Affiliations:** Departamento de Ecología, Genética y Evolución, Facultad de Ciencias Exactas y Naturales, Universidad de Buenos Aires-IEGEBA (CONICET-UBA), Ciudad Universitaria, Pab. 2, Piso 4, Buenos Aires 1426, Argentinalucasleveau@yahoo.com.ar (L.M.L.)

**Keywords:** invasive species, exotic parrots, urban parks, urbanization

## Abstract

**Simple Summary:**

Parrots are among the most globally traded taxa and have successfully invaded urban areas. Studies analyzing alien parrot–habitat relationships are scarce in cities of the Southern Hemisphere. This study aims to determine the habitat characteristics influencing alien parrot species richness and occurrence during the annual cycle in parks in Buenos Aires City. We found that alien parrot species richness was positively associated with tree species richness and a shorter distance to the La Plata River throughout the year. Moreover, parrot richness increased in parks with tree genera like *Eugenia*, *Podocarpus*, *Olea,* and *Washingtonia* during the non-breeding season. During the breeding season, parrot species richness decreased with increasing environmental noise. The relationship between species occurrence and environmental variables changed between seasons. Our findings suggest that exotic parrot richness and presence may be influenced not only by tree diversity and park proximity to green corridors but also by specific exotic tree species providing resources for the parrots. Future urban green space designs should prioritize native tree planting to support local biodiversity.

**Abstract:**

Biological invasions are often one of the main causes of global biodiversity loss. Parrots are among the most globally traded taxa and have successfully invaded urban areas. Studies analyzing alien parrot–habitat relationships are scarce in cities of the southern hemisphere. This study aims to determine habitat characteristics influencing exotic parrot species richness, presence, and composition in urban parks in Buenos Aires City and to analyze variations during breeding and non-breeding seasons. A total of 35 parks were sampled during the breeding season and the non-breeding season, and habitat variables at local and landscape scales were measured. Parrot species richness was positively associated with tree species richness and a shorter distance to the La Plata River throughout the year. During the non-breeding season, parrot species richness increased in parks with a higher abundance of tree genera such as *Eugenia*, *Podocarpus*, *Olea*, and *Washingtonia*. However, during the breeding season, parrot species richness decreased with increased environmental noise. Taxonomic richness was higher during the breeding season. The occurrence of different species and composition depended differentially on each variable, and it varied between seasons. Our findings suggest that exotic parrot richness and presence may be influenced not only by tree diversity and park proximity to green corridors but also by specific exotic tree species providing resources for the parrots. Future urban green space designs should prioritize native tree planting to support local biodiversity over exotic trees that benefit invasive bird species.

## 1. Introduction

Biological invasions are among the main drivers of native biodiversity loss on a global scale [1,2]. In many cases, these invasions result from the intentional or accidental release of illegally traded species, often kept as pets [3]. The trade of wildlife has been directly linked to the introduction of exotic bird species, many of which become invasive [4]. Studies have suggested that propagule pressure (measured as the number of individuals released in a non-native region) is one of the key factors explaining invasion success [5,6]. Once established in the wild, invasive birds become part of the local biotic community and inevitably have an impact on their new environment [7].

Among them, the order Psittaciformes (parrots) ranks among the most globally traded taxa [8], followed by the orders Passeriformes, Falconiformes, and Columbiformes [9]. Across the world, numerous parrot species have successfully established themselves in urban and peri-urban environments [10]. Approximately 10% of the species from the Psittaciformes order have established exotic populations worldwide [11].

The overall impact of exotic psittacids on native communities remains understudied [12], but recent research has shown that its main impact on native wildlife is through a mechanism of interspecific competition [13]. They could directly compete with taxa that utilize cavities for nesting, such as woodpeckers (Aves: Piciformes) or bats (Mammalia: Chiroptera) [12,14], and some species of secondary hole-nesting passerines (Aves: Passeriformes), such as the nuthatches in Belgium [15]. Indeed, a recent study has shown that urban populations of alien psittacids not only use the same cavities as a native species of bat but also exhibit highly aggressive behavior towards them, even leading to their death [16]. Additionally, parrots can act as vectors for human psittacosis and potentially cause damage to crops near urban areas [12,17].

Several factors, including the high availability of food resources and nesting sites provided by a variety of tree species, combined with a low density of natural predators, have contributed to the expansion of exotic parrots in cities [18,19,20]. That lack of natural predators in their invasive range can also increase the reproductive capacity of alien parrot species compared to their native distribution area [21]. In addition to the food availability provided by trees and shrubs, bird feeders provided by humans could also be a determining driver favoring the establishment and occurrence of parrots in urban areas [22]. However, the distribution, abundance, and presence of exotic parrots in urban areas of the Neotropical Region have been poorly explored [20].

A previous study suggests that large expanses of urban green spaces can be likened to natural forest patches, which could favor the establishment of parrots in cities [23]. These natural spaces might include parks and squares with less isolation between them, or larger areas [24]. Furthermore, other researchers have indicated a negative association between noise pollution and bird establishment in urban green areas [12,25], with most studies focusing on Northern Hemisphere countries.

In several urban areas of Argentina, particularly in Buenos Aires City and the Metropolitan Area of Buenos Aires, self-sustaining populations of parrots native to other ecoregions of the country have been present since the 1980s, feeding and reproducing in these new territories [10,26,27]. In Buenos Aires City, 10 species of psittacids have been recorded [28], of which 6 have reproductive populations in the area [29]: the Turquoise-fronted Amazon (*Amazona aestiva*), Nanday Parakeet (*Aratinga nenday*), Yellow-chevroned Parakeet (*Brotogeris chiriri*), White-eyed Parakeet (*Psittacara leucophthalmus*), Maroon-bellied Parakeet (*Pyrrhura frontalis*), and Monk Parakeet (*Myiopsitta monachus*). The Monk Parakeet is the only native species in the region, while the others originate from the Paranaense, Chaco, and Yungas phytogeographic provinces [30,31]. Recent studies carried out near our study area have found that some of these species nest in exotic trees like cypresses (*Cupressus* sp.) and plane trees (*Platanus acerifolia*) [27]. Furthermore, the Turquoise-fronted Amazon, Nanday Parakeet, and Maroon-bellied Parakeet show seasonal variations in their abundance in other cities in the Buenos Aires province, being more abundant during the breeding season [27]. However, the relationship between environmental variables and the presence, taxonomic richness, and composition of exotic parrots in Buenos Aires City has not been analyzed yet.

Understanding the ecology of these exotic species in Buenos Aires City can contribute to developing appropriate management techniques and strategies to minimize the potential impacts of invasion on native species. Therefore, our objective is to analyze exotic parrot–habitat relationships during an annual cycle in Buenos Aires City.

Since the number of bird species generally increases with park size [24,32,33], higher taxonomic richness and occurrence of exotic parrot species are expected in larger parks. This richness and occurrence will also be higher in less isolated green areas [34,35]. Additionally, greater taxonomic richness and occurrence are anticipated in areas near large bodies of water such as the La Plata River [25,36], which could serve as a green corridor providing resources to parrots [37]. On the other hand, since parrots benefit from a variety of tree species that provide food resources and nesting sites, higher taxonomic richness and parrot occurrence are expected in parks with greater vegetation cover and heterogeneity [23,38,39,40]. Some authors have also suggested that disturbance from and the intensity of human activity affect bird species composition [36,41,42,43]. For instance, species richness and occurrence can decrease in sites with higher noise due to reduced vocal communication capacity among species [44,45].

Lastly, we expected that variations in taxonomic richness, occurrences, and species composition would exist between the breeding and non-breeding seasons [27], with a higher presence of parrot species during the breeding season. This is based on citizen science data, which shows that during the breading season the species *Ps. leucophthalmus* has higher occurrence frequencies in Buenos Aires City, with numbers being very low or nonexistent during the non-breeding season [28]. It is also expected that relationships between parrots and environmental variables will differ between the breeding and non-breeding seasons [46]. Parrot–habitat relationships will be more pronounced during the breeding season, as parrots have habitat requirements linked to nesting and feeding. During the non-breeding season, parrots disperse and primarily have feeding requirements, causing their habitat relationships to be less pronounced or more related to factors such as distance from the nearest green space [47,48].

## 2. Materials and Methods

### 2.1. Species Analized

Five parrot species native to South America (but not from the study area) have been analyzed (Table 1). In general terms, all species are found in Brazil, Paraguay, and Bolivia, among other countries, reaching only as far as the northern region of Argentina. They all feed on fruits, seeds, and flowers and generally prefer wooded habitats, gallery forests, and in some cases, open environments. The average body size of the five species is 30.6 cm. *Am. aestiva* is the only species with a Near Threatened conservation status, while the others are of Least Concern [49]. However, even though these species are currently classified as Least Concern, this category could be subject to change in the future because all species face strong pressure from capture for the pet trade and habitat loss and degradation [49].

### 2.2. Study Area

**Figure 1:**
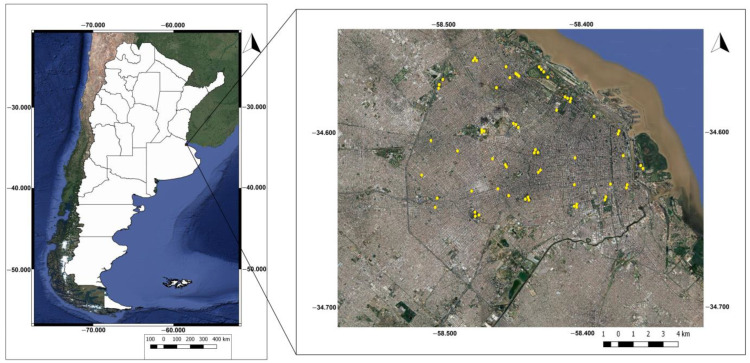
Political map of the Argentine Republic (**left**) and satellite image (Google Earth) of Buenos Aires City (**right**), showing the location of sampling points in yellow.

This study was carried out in Buenos Aires City (34°36′14″ S, 58°26′54″ W), which covers an area of 203 km^2^ and has a population of 3,120,612 inhabitants [55], making it the largest and most populated city in Argentina. Situated in the central–eastern region of the country, it borders the La Plata River to the east, is surrounded to the south, north, and west by the Buenos Aires province (Figure 1) and is encircled by 24 districts that constitute the Greater Buenos Aires metropolitan area, where approximately 40% of the Argentine population resides.

Buenos Aires City is predominantly located within the Pampa region [56,57], whose native landscape was once characterized by grasslands and xerophilous forest formations but was later replaced by agricultural systems and plantations of exotic trees [58]. With the subsequent population growth, this landscape transformed into an urban environment [57,59]. Currently, the Buenos Aires City landscape consists of a matrix of paved streets and buildings, with green patches formed by parks and other green spaces [60]. These green spaces are highly homogeneous in terms of habitat structure and are composed of grassy areas and sections with trees and some shrubs. Exceptions include areas like the “bosques de Palermo” (Palermo Parks), which feature water bodies and consequently a greater richness of animal species [36]. Approximately 10% of the total city area is classified as green space [61].

### 2.3. Selection of Sampling Sites

A total of 35 urban green areas (parks) in Buenos Aires City were selected using Google Earth Pro^®^ software (version 7.3.6.9345), with the aim of representing a broad environmental gradient. Within these areas, different numbers of sampling points were selected based on the size of the area (0 to 5 ha.: 1 point, 5 to 8 ha.: 2 points, 8 to 18.3 ha.: 3 points, >18.3 ha.: 4 points). These points were spaced at least 200 m apart and at least 50 m from the edge of the green area to ensure independence between sampling units [62] (Figure 1).

The selection of parks was initially conducted in a stratified manner, considering socioeconomic level and urban landscape structure. This selection was later adjusted based on logistical and safety considerations for the sampler. Consequently, certain areas initially chosen had to be modified due to opening hours, inaccessibility, and safety concerns.

### 2.4. Bird Counts

Each point was visited six times during the year 2022. The first sampling period took place from 22 March to 1 April, the second from 12 May to 26 May, the third from 18 July to 30 July, the fourth from 11 September to 23 September, the fifth from 1 November to 9 November, and the sixth from 16 December to 26 December. In other words, the first three sampling periods occurred during the Southern Hemisphere’s autumn and winter seasons, while the last three took place during the Southern Hemisphere’s spring and summer seasons. The autumn–winter samplings corresponded to the non-breeding season of the birds, whereas the spring–summer samplings corresponded to the breeding season [63].

For each point and each visit, every species of parrot was recorded through direct observation or vocalization. The abundance of each species was noted if possible, indicating whether the bird was seen and/or heard within a radius greater than 50 m and a radius smaller than 50 m, during a 5 min period, and within a time frame no later than 4 h after sunrise. Nikon Aculon 12 × 50 binoculars were used to locate birds, whereas a Xiaomi Redmi^®^ smartphone was used to record vocalizations and confirm species identity, if necessary, through the Xeno-Canto platform [64]. Bird observations and bird counts were consistently conducted by the same observer (SMS), excluding days with adverse weather conditions (rain, fog) that could affect bird detection [62]. NPC assisted with environmental variables counts and field annotations.

### 2.5. Environmental Variables

For each park, variables were determined at the local scale within each park and at the landscape scale outside the parks. Local-scale variables included area, tree coverage, tree diversity, tree species richness, pedestrian traffic, and environmental noise. The area in hectares (“Park size” hereafter) of each park or square was estimated using QGIS^®^ 2.8.14 geographic information system software through polygon tracing. Tree coverage (“Tree Cover” hereafter) was estimated using QGIS^®^ 2.8.14 software. The percentage of Tree Cover for each green area was delineated using polygons on satellite images of tree canopies (Google Earth images) within the parks, and the polygon’s value (in ha.) was divided by the total area of the park. Tree diversity (“H_tree” hereafter) and tree species richness (“S_tree” hereafter) were calculated using data (number of individuals) provided by the Buenos Aires City green spaces dataset [61] for each green area. For H_tree, the Shannon diversity index [65] was calculated using the “diversity” function from the “vegan” package in R [66]. S_tree was the total number of tree species in each green area, and it was calculated with the “specnumber” function from the vegan package in R [66].

Tree genera composition was analyzed through a Non-Metric Multidimensional Scaling (NMDS) (Figure S1, Supplementary Materials) analysis to ordinate tree genera composition among parks using the “metaMDS” function from the vegan package in R [66]. An NMDS is a statistical technique used for reducing the dimensionality of multivariate data and represents it in a lower-dimensional space (usually two or three dimensions) while preserving the pairwise dissimilarities or distances between data points [67], which makes it useful for visualizing and exploring patterns, similarities, and differences among data points. Therefore, we used this technique to summarize the composition of tree genera in the parks and extract that information to observe associations with the parrot species richness and occurrence variables through Generalized Lineal Models (GLM). Abundance data for trees were derived from the Buenos Aires City green spaces dataset [61]. Then, parks were ordinated according to the Bray–Curtis dissimilarity index. Subsequently, scores for two axes (Tree_comp1 and Tree_comp2 hereafter) were calculated from this ordination, representing the location of each sample in the space based on its dissimilarity to the objects or samples (as the case may be) included in the analysis. This was performed using the “scores” function from the vegan package in R [66]. A graphical representation of the ordination was created to qualitatively visualize the arrangement of tree genera in the parks (Figure S1, Supplementary Materials). These score values obtained for each park and each axis were used as explanatory variables in the GLM (see “Statistical Analysis,” next page) to associate parrot richness and composition with tree composition.

Landscape-scale variables included distance to La Plata River and isolation. Distance to La Plata River (“Distriv” hereafter) was estimated using the “ruler” tool in Google Earth Pro^®^ software, measuring the distance in meters from the closest eastern vertex of each park to the nearest point on the La Plata River. Isolation between sites (“Isol” hereafter) was estimated using R software [68] and the Buenos Aires City government dataset [61] as the distance in meters between the edge of each park and the nearest green area of at least 1 hectare in size with the presence of trees. Isol represents how far each green area in our study is from a nearby wooded green area of at least 1 ha extension.

Furthermore, anthropogenic disturbance variables were recorded for each point simultaneously during bird counts, such as the number of pedestrians (pedestrians/5 min.) within a 50 m radius and ambient noise (dB), measured for 1 min at the beginning and end of each sampling using the “Sound Meter” application [69] with a Xiaomi Redmi^®^ smartphone (“Pedest” and “Db” hereafter). For each park, average values were obtained from all points and visits in each season.

### 2.6. Variables of Parrot Diversity and Occurrence

The richness of the parrot species was the total number of parrot species in each park during each season (“S” hereafter) and it was calculated using the specnumber function from the vegan package in R [66].

Due to the biology of the species in question, which are often noisy and, in most cases, easier to detect aurally than visually, and being species that often form large flocks, the decision was made to work with presence–absence data rather than abundances. This choice was made to avoid the high likelihood of underestimated abundance data (i.e., hearing a few individuals while being aware that there could be many more around them not vocalizing, especially when flocks are sheltered in tree canopies, for instance). Therefore, parrot species richness was used instead of their diversity, and presence/absence data (occurrence or occupancy) were used to calculate species composition and the relationship between each species and environmental variables.

### 2.7. Statistical Analysis

A correlation analysis was performed between the explanatory variables using the Pearson coefficient with the “cor” function in R [68]. We found a correlation value greater than 0.7 between S_tree and diversity of trees (H_tree), and between Tree_comp1 and H_tree, indicating a high correlation [70]. Therefore, we chose the variable that had a stronger relationship with the response variable of the specific model.

Subsequently, the “scale()” function in R was used to standardize each variable so that they had a mean of zero and a standard deviation of one. This standardization allowed for the simultaneous inclusion of these variables in the models. Separate analyses were conducted for the breeding and non-breeding seasons.

The relationship between parrot species richness and composition and environmental variables was assessed through a GLM, using a Poisson error structure for richness and a binomial structure for species occurrence (presence/absence data). For model selection, a “forward” selection approach was performed using the “add1” function in R, which iteratively evaluates the effect of adding variables that significantly improve the model until a final model is reached. Starting with a null model that includes only the random intercept, one explanatory variable was added at a time, and its effect was evaluated using the Chi-squared test. Only variables that were significant (*p* < 0.05) and had a lower Akaike Information Criterion (AIC) value were included in the model.

For all models related to taxonomic richness and species occurrence, a Likelihood ratio test was performed comparing the final models with a null model (a simple model including only a random intercept), testing that the proposed model was significantly better (*p* < 0.05) than the null model in terms of fit and predictive capability.

Model assumptions were assessed using the “simulateResiduals” function from the DHARMa package in R [71], ensuring no deviations from the assumptions both analytically and visually. A significance level of α = 0.05 was used for all analyses. When evaluating the assumptions of the taxonomic richness model, it was found that the assumption of equidispersion was not met. Therefore, the process was repeated using a Conway–Maxwell–Poisson model through the “glmmTMB” function [72].

For species occurrence models, only a model for the White-eyed Parakeet (*Ps. leucophthalmus*) during the breeding season was obtained due to the limited data available for this species in the non-breeding season.

To compare the parrot species richness between breeding and non-breeding seasons, a paired *t*-test was conducted in R using the “t.test” function. The assumption of normality for the paired *t*-test was checked using a Shapiro–Wilk test in R, while the homogeneity of variances was assessed using a Levene test in R. A contingency table was created to compare species occurrences between seasons, examining the potential independence of parrot occurrence across seasons by comparing the observed and expected frequencies. The assumptions and Yate’s correction for 2 × 2 tables were assessed prior to conducting the analysis of the contingency table.

For the analysis of species composition of parrots, a Non-Metric Multidimensional Scaling (NMDS) was performed using the metaMDS function from the vegan package in R [66]. This analysis ordered the occurrence of parrot species in the sampled sites using a Jaccard dissimilarity index based on presence/absence data. The recorded environmental variables for each site were fitted to this ordination using the “envfit” function from the vegan package [66]. The adjusted ordination was graphed only for those environmental variables that showed statistical significance (*p* < 0.05). This analysis considered only sites where at least one species was present, excluding green areas where no species were recorded.

## 3. Results

A total of five species were recorded, of which *B. chiriri* and *Py. frontalis* were the most frequent during the non-breeding season, and *Ps. leucophthalmus* and *B. chiriri* were the most frequent during the breeding season.

During the non-breeding season, the taxonomic richness model included the explanatory variables Park size, Distriv, S_tree, and the second axis of the NMDS of tree composition (Tree_comp2), of which the latter three were significant (*p* < 0.05), and Park size was not significant (*p* = 0.272) (Table 2A) (Chi-sq = 32.22, df = 4, *p* < 0.001).

**Figure 2:**
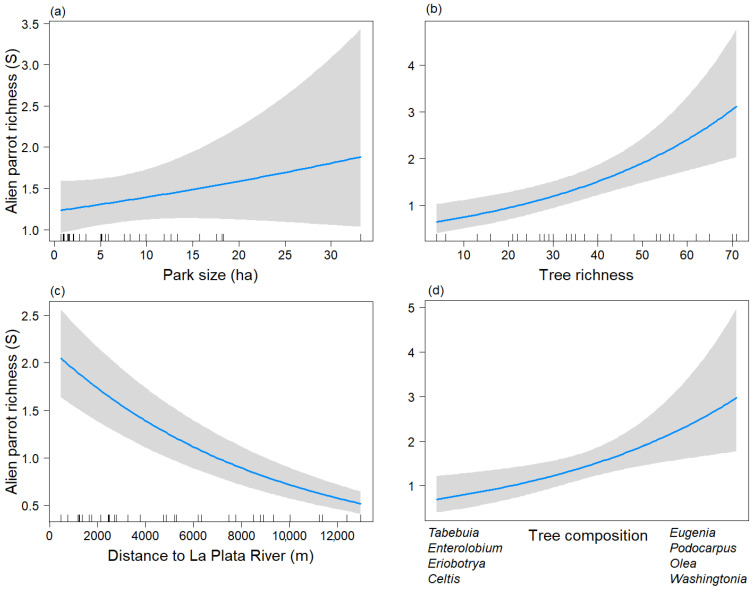
Predictive plot of the linear relationship between the taxonomic richness of exotic parrots (S) and the model’s explanatory variables for the non-breeding season, along with its corresponding confidence 95% band (gray). (**a**) Park size; (**b**) Tree richness; (**c**) Distance to La Plata River; (**d**) Tree composition.

S_tree and the Tree_comp2 showed a positive relationship with the taxonomic richness of parrots (Figure 2b,d). Parrot species richness was higher in parks dominated by the genera *Eugenia, Podocarpus, Olea,* and *Washingtonia*, and lower in parks dominated by the genera *Tabebuia, Enterolobium, Eriobotrya,* and *Celtis*. On the other hand, the distance to the La Plata River variable displayed a negative association with the taxonomic richness of exotic parrots (Figure 2c). Therefore, parrot species richness decreased in parks isolated from the La Plata River. Park size showed a positive association with taxonomic parrot species richness, but it was statistically non-significant (*p* = 0.272 (Figure 2a).

During the breeding season, the final model for parrot richness included the variables Park size, Db, Distriv, and S_tree, with Park size again being non-significant (*p* = 0.051) and the other three variables being significant (*p* < 0.05) (Table 2B) (Chi-sq = 30.82, df = 4, *p* < 0.001).

**Figure 3:**
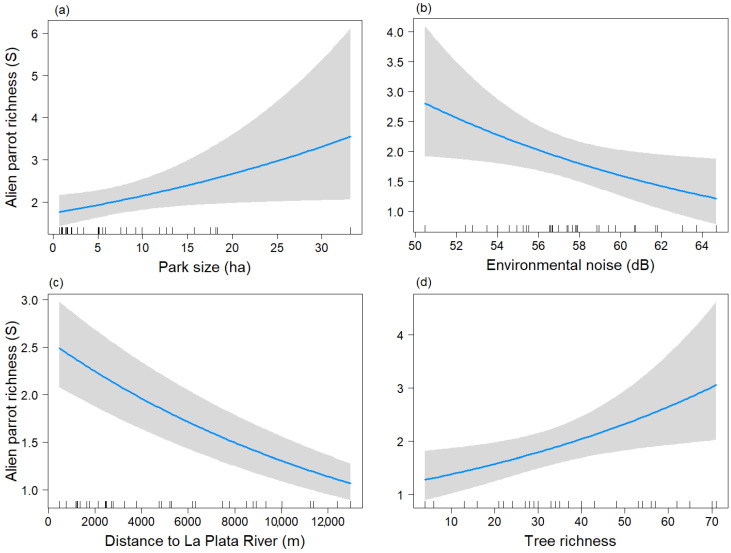
Predictive plot of the linear relationship between parrot species richness (S) and the model’s explanatory variables for the breeding season, along with its corresponding confidence 95% band (gray). (**a**) Park size; (**b**) Environmental noise; (**c**) Distance to La Plata River; (**d**) Tree richness.

The variable Park size tended to show a significant positive relationship (*p* = 0.051) with the parrot species richness (Figure 3a), similar to the non-breeding season. Parrot species richness was negatively related to Db and Distriv (Figure 3b,c). The variable “S_tree” showed a positive association with parrot species richness (Figure 3d).

**Figure 4:**
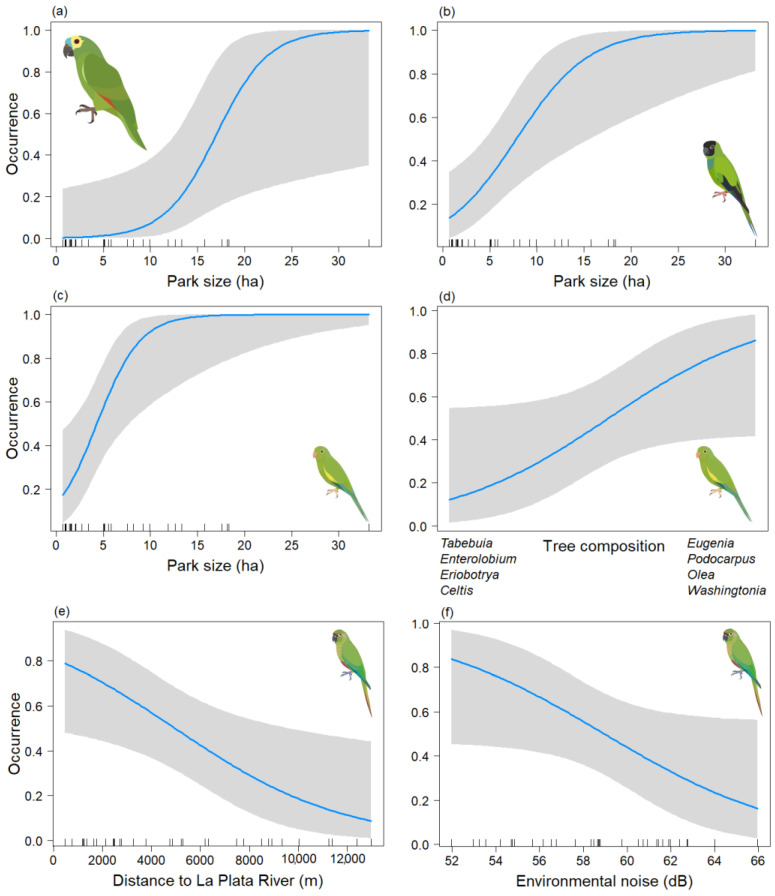
Predictive plot of the relationship between the occurrence of the psittacid species and the model’s explanatory variables for the non-breeding season, along with its corresponding confidence band (gray). (**a**) *Am. aestiva*; *(***b**) *Ar. nenday*; (**c**,**d**) *B. chiriri*; and (**e**,**f**) *Py. frontalis*.

In the analysis of the psittacid species occurrence during the non-breeding season, final models were obtained for *Am. aestiva* and *Ar. nenday,* both of which included only the Park size variable. In both cases, this variable was significant (*p* < 0.05) (Table 3a,b) (Chi-sq = 13.57, df = 1, *p* < 0.001; Chi-sq = 13.62, df = 1, *p* < 0.001, respectively), showing a positive association between the size of the area and the presence of the species *(*Figure 4a,b). In the case of *B. chiriri,* a model was derived that depended on both the Park size and axis two of the NMDS tree composition (Tree_comp2), but only the former variable was found to be significant (*p* < 0.05) (Table 3c) (Chi-sq = 17.6, df = 2, *p* < 0.001). This indicates a positive association between the presence of the species and larger areas (Figure 4c). There also appears to be a positive trend with the tree composition of the genera *Eugenia*, *Podocarpus, Olea*, and *Washingtonia* (Figure 4d), but this relationship is not statistically significant (*p* = 0.060). For *Py. frontalis* a model was derived that depended on the Db and Distriv variables, but only the distance to the river was found to be significant (*p* < 0.05) (Table 3d) (Chi-sq = 10.05, df = 2, *p* < 0.001). This indicates a negative association between greater distance from the river and the presence of the species (Figure 4e) and a negative trend with environmental noise (Figure 4f). However, the latter variable was not statistically significant (*p* = 0.058). For *Ps. leucophthalmus*, only three data points were obtained for this season, so its occurrence could not be effectively modeled.

**Figure 5:**
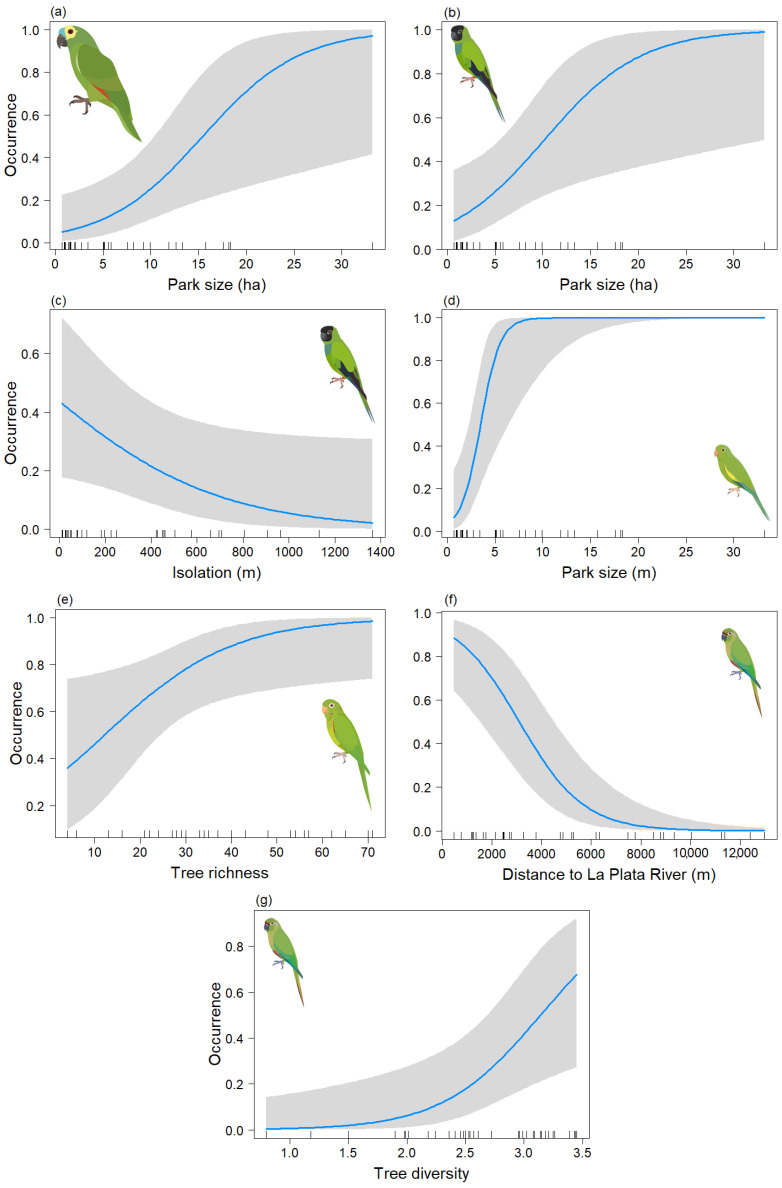
Predictive plot of the relationship between the occurrence of the psittacid species and the explanatory variables of the model for the breeding season, along with its corresponding confidence band (gray). (**a**) *Am. aestiva*; (**b**,**c**) *Ar. nenday*; (**d**) *B. chiriri*; (**e**) *Ps. leucophthalmus*; and (**f**,**g**) *Py. frontalis*.

For the breeding season, models were derived for *Am. aestiva* and *B. chiriri* species that depended solely on the Park size, and in both cases, this variable was significant (*p* < 0.05) (Table 4a,c) (Chi-sq = 9.44, df = 1, *p* < 0.001; Chi-sq = 29.5, df = 1, *p* < 0.001, respectively). Positive relationships were observed in both cases between an increase in area and the occurrence of the species (Figure 5a and Figure 5d, respectively). For *Ar. nenday*, a model dependent on Park size and isolation was obtained, but only the former was significant (*p* < 0.05) (Table 4b) (Chi-sq = 14.87, df = 2, *p* < 0.001). A positive association between the species occurrence and an increase in area was also observed (Figure 5b), as well as a negative (though statistically insignificant) association between occurrence and increased isolation (Figure 5c). For *Ps. leucophthalmus*, a model dependent on tree species richness was derived, and this variable was significant (*p* < 0.05) (Table 4d) (Chi-sq = 6.59, df = 1, *p* < 0.001), showing a positive association with the occurrence of the species (Figure 5e). Lastly, for *Py. frontalis*, a model dependent on distance to La Plata River and tree diversity was obtained, and both variables were significant (*p* < 0.05) (Table 4e) (Chi-sq = 21.44, df = 2, *p* < 0.001). This showed a negative association between the occurrence of the species and the distance to the river (Figure 5f) and a positive association between the occurrence of the species and tree diversity (Figure 5g).

**Figure 6:**
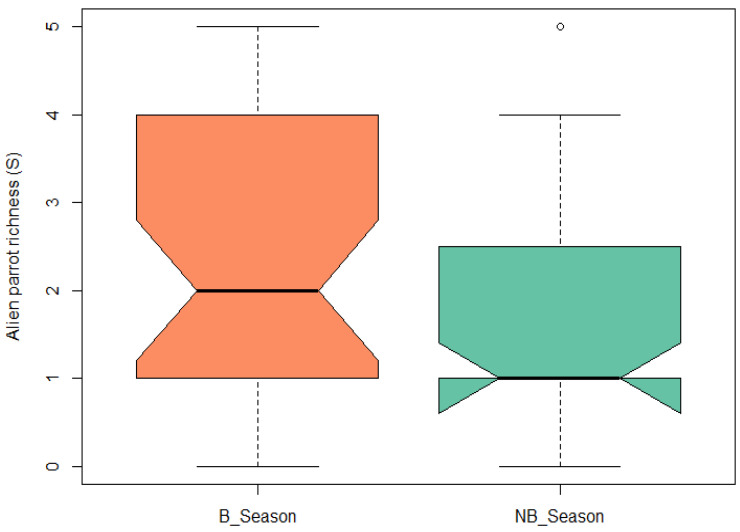
Boxplot of exotic parrot species richness for the breeding season (B_Season) and non-breeding season (NB_Season). Central black line represents the median of the data. Boxes represent the interquartile range (IQR), which is the central 50% of the data. Whiskers represent the dispersion of data outside the IQR.

Regarding the comparison of parrot species richness between the breeding and non-breeding seasons, a paired *t*-test revealed that the difference was significant (t = 3.57, df = 34, *p*-value = 0.001). The richness was greater during the reproductive season (Figure 6).

Furthermore, it was observed that for *Am. aestiva, Ar. nenday*, *B. chiriri,* and *Py. frontalis* there were no significant differences in their presence in green areas between seasons (Chi-sq = 0.97, df = 1, *p*-value = 0.325; Chi-sq = 0.06, df = 1, *p*-value = 0.806; Chi-sq = 0.06, df = 1, *p*-value = 0.809; and Chi-sq = 1.47, df = 1, *p*-value = 0.23, respectively). However, the presence of *Ps. leucophthalmus* showed significant differences between seasons (Chi-sq = 33.6, df = 1, *p*-value < 0.01), with a higher occurrence during the reproductive season.

**Figure 7:**
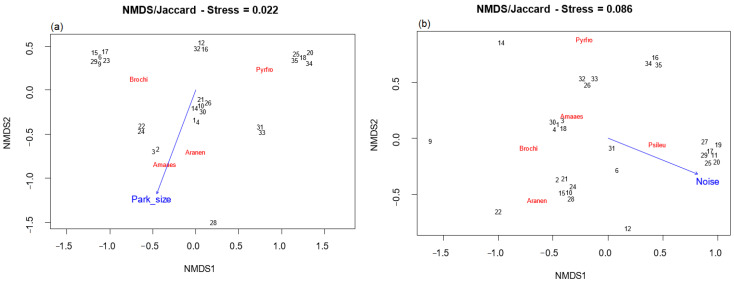
NMDS ordination analysis of exotic parrot species composition and habitat variables during the (**a**): non-breeding season and (**b**): breeding season. The vector indicates the direction in which the variable increases. Amaaes: *Amazona aestiva*, Aranen: *Aratinga nenday*, Brochi: *Brotogeris chiriri*, Psileu: *Psittacara leucophthalmus*, and Pyrfro: *Pyrrhura frontalis*. The numbers (in black) represent each of the parks. Black numbers (parks) were manually separated for better visualization because they overlapped in the original plot.

For the non-breeding season, the axes of NMDS for specific parrot composition were significantly associated with Park size (*p* < 0.05). There was a positive relationship between the area and the species *Am. aestiva* and *Ar. nenday* and a negative relationship with the species *Py. frontalis* (Figure 7a).

On the other hand, for the breeding season, Db (environmental noise) was the only variable significantly associated (*p* < 0.05) with the axes of NMDS for specific parrot composition. It showed a positive association with the species *Ps. leucophthalmus* and a negative association with the species *Am. aestiva, Ar. nenday, B. chiriri,* and *Ps. frontalis* (Figure 7b).

None of the models obtained with the forward selection included (statistically significant) variables of pedestrian traffic, isolation, or tree cover.

## 4. Discussion

The results obtained indicated that parrot species richness and species presence were related to tree diversity and proximity to biological corridors (influenced by the La Plata River basin), especially during the non-breeding season. Furthermore, human disturbance could influence parrot species richness during the breeding season.

Consistent with our hypotheses and predictions, during both seasons exotic parrot species richness was greater in green areas with higher tree species richness and proximity to La Plata River (associated with a green corridor adjacent to the river). On one hand, the positive relationship with tree species richness suggests that greater habitat heterogeneity benefits parrot species richness, aligning with other studies on urban birds where taxonomic diversity was positively associated with tree species richness [39]. On the other hand, the proximity to the river implies closeness to a biocorridor composed of a continuum of natural reserves and connected green spaces, as discussed by Haene [39]. This is in line with other research where urban bird richness increased in locations near large bodies of water [25,36].

During the non-breeding season, a positive relationship was observed between parrot species richness and exotic tree genera that could provide food and shelter for the parrots, such as *Eugenia* and *Podocarpus* (berry-bearing fruits) as well as *Olea* and *Washingtonia* (drupe-bearing fruits). These findings align with other studies indicating that exotic parrot species in other regions of the Buenos Aires province preferentially utilize exotic trees for feeding and nesting rather than native trees [27]. Another study in temperate European cities has also shown that the occurrence and establishment of opportunistic granivorous–frugivorous parrot species were positively associated with the presence of habitats with higher fruit and seed diversity during the non-breeding season [22], also aligning with our results.

Other researchers have found that genera like *Podocarpus* [73,74], *Olea* [75,76], and *Washingtonia* [19,77] were positively associated with the presence of some psittacid species. This is due not only to the utilization of their fruits and seeds for food but also for nesting, especially in palm species like *Washingtonia*, where the type of trunk and dry leaf attachments create cavities suitable for nesting [19]. A positive trend was also found between the abundance of *Washingtonia* and the occurrence of *B. chiriri* during the non-reproductive season, suggesting the potential provision of food resources as its fruits grow during autumn in Buenos Aires (SMS, personal observation).

During the breeding season, a negative relationship between taxonomic richness and environmental noise was observed. This might suggest that these species seek quieter areas during the breeding season, similar to what has been observed in other studies of urban birds, where bird diversity decreases with higher levels of anthropogenic disturbance [41,43]. For instance, sites with lower noise levels could enhance communication among individuals, as discussed in Dooling and Popper [44]. This could potentially favor the richness of parrots in less disturbed parks during the reproductive season.

During the reproductive season, although the taxonomic richness of exotic parrots showed a non-significant association with the area (*p* = 0.051), the obtained *p*-value suggests that the relationship was close to being statistically significant. This could indicate that in this season, the area is important for the taxonomic diversity of parrots, consistent with other studies on urban birds [24,32,33,77]. The positive relationship with park size might indicate greater resource availability in larger areas compared to smaller ones, partially verifying our proposed hypotheses and predictions.

For the species occurrence analysis, irrespective of the season, *Am. aestiva*, *Ar. nenday*, and *B. chiriri* displayed positive associations between their presence and park size. This result is consistent with other studies showing that park size positively influences bird diversity and presence in urban areas [24,32,33,77]. Authors like Davis et al. [23] suggest that expansive green spaces in cities resemble forest patches, favoring parrot occurrence. Given that these species often fly in numerous flocks, the relationship makes sense: larger parks offer more opportunities for the flock to find space and resources, thus determining species presence, aligning with our hypothesis that larger areas are positively associated with species occurrence.

In the case of the *Ps. leucophthalmus*, its presence was only detected during the reproductive season. This pattern, also recorded in citizen science data [28], suggests that the species migrates from the city to other sites during autumn and winter. Similar to other studies on psittacids in urban environments, these local migrations from cities to adjacent areas could be due to annual fluctuations in resources and nesting sites between locations [78,79,80]. Additionally, in urban environments in Brazil, the abundance of *Brotogeris versicolorus* is negatively related to that of *Ps. leucophthalmus*, implying interspecific competition could affect temporal variations in the latter species in cities [81].

The occurrence of *Ps. leucophthalmus* during the breeding season was positively associated with areas with higher tree species richness, suggesting this species specifically selects green areas with greater habitat heterogeneity for reproduction. Geary et al. [82] found that the occurrence of another congeneric species, *Psittacara chloropterus*, was positively associated with tree species richness in parks in urban zones in the Dominican Republic. This is due to the increased availability of food resources and nesting sites.

*Py. frontalis* showed a positive association with parks near the biological corridor of the La Plata River during both non-breeding and breeding seasons. This suggests that these areas are utilized for both feeding and reproduction. During the breeding season, a positive relationship with tree diversity was also observed, implying that heterogeneous sites can provide various feeding and nesting locations, as discussed in Shwartz et al. [39] for urban birds and Geary et al. [82] where tree species richness in parks favors parrot occurrence. For the non-breeding season, the occurrence of this species seemed to be negatively related to environmental noise, as suggested in other works where bird presence in parks is negatively correlated with environmental noise [41,43].

None of the models obtained included the variables of pedestrian traffic, isolation, or tree cover (as they were not statistically significant), which is unexpected given that several studies with birds indicate that higher pedestrian traffic decreases diversity and the likelihood of bird occurrence [36,41,42,43].

Nor was a significant relationship found between vegetation cover and patch isolation [35]. However, for *Ar. nenday,* although its occurrence did not yield significance (*p* = 0.053) regarding patch isolation, the obtained *p*-value is close to being statistically significant, partially confirming our hypothesis of occurrence in sites with lower isolation from each other during the reproductive season.

Parrot species richness was higher during the breeding season compared to the non-breeding season, probably due to the arrival of the *Ps. leucophthalmus* during the reproductive season. This agrees with the results of Ibañez et al. [27], who found the species was more abundant during the non-reproductive season in a nearby locality from Buenos Aires City, suggesting species migration.

During the non-breeding season, the axes of NMDS for specific composition were significantly associated with the park area, whereas for the breeding season, they were associated with environmental noise. This indicates that species composition presented distinct patterns of association with habitat and environmental variables for each season. Therefore, the significant association between parrot species and green area size could indicate the availability of resources is more important during the non-breeding season. On the other hand, the significant association between parrot species and noise during the breeding season could indicate that the facilitation of vocal communication is more important during breeding.

Our results confirmed the findings of Leveau and Leveau [46], where the relationships between bird species and habitat variables varied between the breeding and non-breeding seasons. Due to anthropogenic disturbance potentially affecting intraspecific communication among individuals, it is expected that during the reproductive season most species will associate with less noisy areas, as discussed in other studies on birds in cities [44,45]. Additionally, since during the non-breeding season, the relationships between birds and habitat are less restricted, species tend to disperse more [47,48], and larger areas can facilitate species establishment in the location, as they are easier to detect than smaller areas during dispersal.

## 5. Conclusions

Based on the results obtained the presence of trees with food resources and proximity to biological corridors such as the La Plata River could be important factors for the establishment of a higher number of invasive parrot species in parks and squares in Buenos Aires City, especially during the non-breeding season. Additionally, ambient noise could also influence the richness of invasive parrots during the breeding season.

Regarding the occurrence of each individual parrot species, it was observed that each one has different relationships with the studied habitat and environmental variables, which would allow them to exploit different resources and coexist.

The species composition showed different patterns of association with habitat and environmental variables for each season. Our results suggest that during the non-breeding season, species disperse, and the park area may favor establishment in the site during dispersion. During the breeding season, other factors such as communication between individuals might be relevant for mate searching.

We recommend that future green area design projects in Buenos Aires should adopt policies and practices that promote the planting of native tree species from the region (favoring local biodiversity), instead of exotic species that provide food and nesting resources for invasive birds. We also encourage future research projects to study the competitive relationships of these parrot species with the local wildlife in Buenos Aires City or conflicts with human societies.

## Figures and Tables

**Table 1:** Summary of size, native range, habitat preferences, and diet of parrot species studied.

Species	Size (cm)	Native Range	Habitat Preferences	Diet	Sources
*Amazona* *aestiva*	37	Brazil, Bolivia, Paraguay, and N Argentina.	Wooded habitats, gallery forest, and palm groves.	Fruits, seeds, and flowers.	[50]
*Aratinga* *nenday*	30	Bolivia, Brazil, and N Argentina.	Gallery forest, deciduous forest, and palm stands.	Seeds, fruits, nuts, berries, and flowers.	[51]
*Brotogeris* *chiriri*	23	Bolivia, Brazil, Paraguay, and N Argentina.	Open woodlands, savannas, subtropical forest, gallery forest, and towns.	Seeds, nectar, fruits, and blossoms.	[52]
*Psittacara* *leucophthalmus*	35	Colombia, Ecuador, Perú, Brazil, Venezuela, Guianas, Bolivia, Paraguay, N Uruguay, and N Argentina.	Forest edge and adjacent savanna, scrub forest, secondary growth, deciduous or gallery woodland, palm groves, flooded forest, opening in rainforest, also mangroves.	Fruit, blossoms, grass seeds, and even insects.	[53]
*Pyrrhura frontalis*	28	Brazil, Paraguay, NE Argentina, and N Uruguay.	Humid and dry forest, gallery woodlands, partly cleared and thinned areas, and even cities.	Nuts, fruits, seeds, and flowers.	[54]

**Table 2:** Final models assessing the relationship between exotic parrot species richness and the environmental variables during non-breeding (A) and breeding seasons (B) in Buenos Aires City. Park size: area (ha), Distriv: distance to La Plata River (m), S_tree: tree species richness, Tree_comp2: axis two of tree NMDS in parks, Db: environmental noise (Db). CI: confidence intervals.

**(A) Non-Breeding Season**			
**Predictors**	**Estimates**	**CI**	***p*-Value**
Intercept	1.33	1.07–1.65	0.009
Park size	1.10	0.93–1.30	0.272
Distriv	0.67	0.54–0.82	<0.001
S_tree	1.54	1.23–1.93	<0.001
Tree_comp2	1.42	1.12–1.80	0.004
**(B) Breeding Season**			
**Predictors**	**Estimates**	**CI**	***p*-Value**
Intercept	1.95	1.64–2.32	<0.001
Park size	1.17	1.00–1.37	0.051
Distriv	0.78	0.65–0.93	0.005
S_tree	1.27	1.05–1.55	0.016
Db	0.83	0.70–0.98	0.026

**Table 3:** Final binomial models assessing the relationship between parrot species occurrence and environmental variables during the non-breeding season in Buenos Aires City. CI: confidence intervals and Odds Ratios: ratio of the odds of success in an event (species occurrence) to the odds of failure in the event (species absence).

Species	Predictors	Odds Ratios	CI	*p*-Value
(a) *Am. aestiva*	Intercept	0.02	0.00–0.44	0.013
	Park size	13.92	1.24–156.66	0.033
(b) *Ar. nenday*	Intercept	0.73	0.31–0.74	0.479
	Park size	6.51	1.74–24.32	0.005
(c) *B. chiriri*	Intercept	2.82	0.83–9.58	0.096
	Park size	23.41	2.16–253.80	0.010
	Tree_comp2	2.49	0.96–6.44	0.060
(d) *Py. frontalis*	Intercept	0.92	0.42–1.98	0.828
	Distriv	0.34	0.14–0.85	0.021
	Db	0.44	0.19–1.03	0.058

**Table 4:** Final binomial models for the occurrence of exotic parrots on a logit scale for breeding season. CI: Confidence intervals and Odds Ratios: ratio of the odds of success in an event (species occurrence) to the odds of failure in the event (species absence).

Species	Predictors	Estimates	CI	*p*-Value
(a) *Am. aestiva*	Intercept	0.17	0.06–0.52	0.002
	Park size	4.18	1.35–12.94	0.013
(b) *Ar. nenday*	Intercept	0.52	0.21–1.29	0.157
	Park size	4.24	1.25–14.36	0.020
	Isolation	0.39	0.14–1.03	0.058
(c) *B. chiriri*	Intercept	20.80	1.07–404.09	0.045
	Park size	1160.58	7.44–181,029.01	0.006
(d) *Ps. leucophthalmus*	Intercept	5.05	1.70–14.97	0.004
	S_tree	3.75	1.11–12.70	0.033
(e) *Py. frontalis*	Intercept	0.19	0.05–0.79	0.022
	Distriv	0.06	0.01–0.41	0.004
	H_tree	4.72	1.15–19.44	0.031

## Data Availability

The datasets generated and/or analyzed during the current study are available upon request to the corresponding author.

## Supplementary Materials

The following supporting information can be downloaded at: https://www.mdpi.com/article/10.3390/ani13213426/s1, Figure S1: NMDS ordination analysis for the tree genera composition among parks in Buenos Aires City. The numbers (in black) represent each of the parks.
