# Supplementary material for: What Drives the Alien Parrot Richness and Occurrence in Urban Green Spaces along the Annual Cycle in Buenos Aires City, Argentina?
Source: Animals (Basel). 2023 Nov 6;13(21):3426. doi: 10.3390/ani13213426 (PMC10648644; doi:10.3390/ani13213426)
Supplement: Supplementary file 1 [file animals-13-03426-s001.zip › animals-2659580-supplementary.pdf]

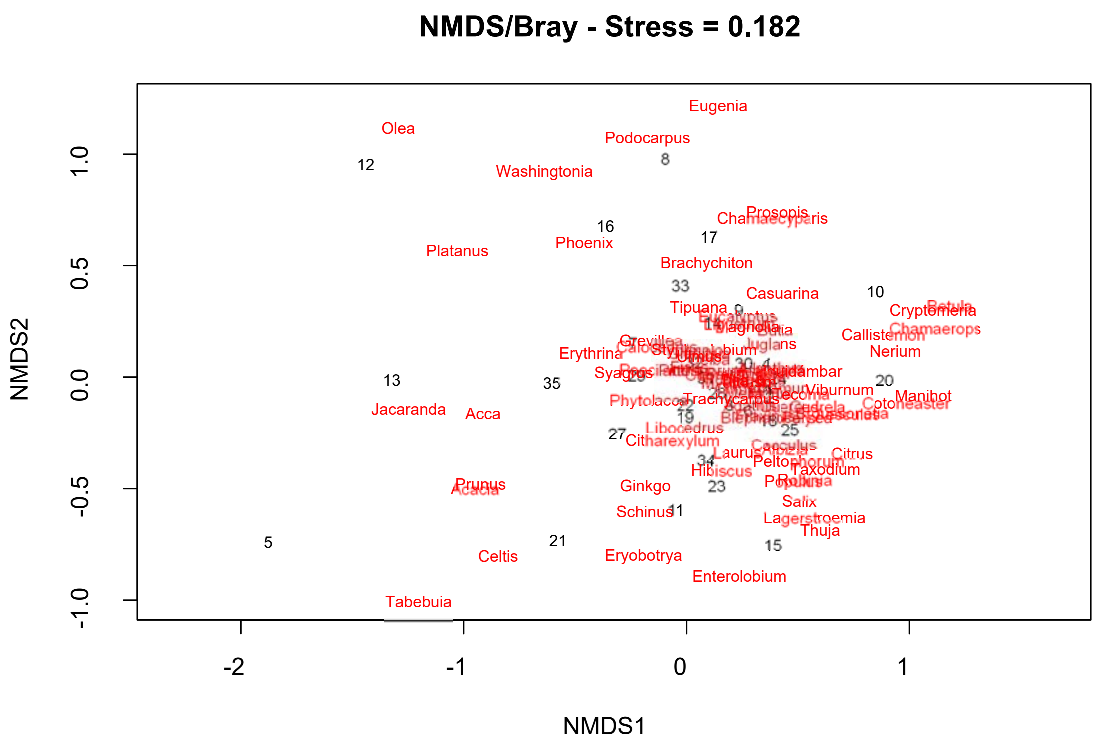

Figure S1. NMDS ordination analysis for the tree genera composition among parks in Buenos Aires City. The numbers (in black) represent each of the parks.
